# Molecular imaging of aberrant crypt foci in the human colon targeting glutathione S-transferase P1-1

**DOI:** 10.1038/s41598-017-06857-x

**Published:** 2017-07-26

**Authors:** Naoki Muguruma, Koichi Okamoto, Tadahiko Nakagawa, Katsutaka Sannomiya, Shota Fujimoto, Yasuhiro Mitsui, Tetsuo Kimura, Hiroshi Miyamoto, Jun Higashijima, Mitsuo Shimada, Yoko Horino, Shinya Matsumoto, Kenjiro Hanaoka, Tetsuo Nagano, Makoto Shibutani, Tetsuji Takayama

**Affiliations:** 1 Department of Gastroenterology and Oncology, Tokushima University Graduate School of Biomedical Sciences, Tokushima, 770-8503 Japan; 2 Department of Digestive and Pediatric Surgery, Tokushima University Graduate School of Biomedical Sciences, Tokushima, 770-8503 Japan; 3 R&D Group, Olympus Corporation, Hachioji, Tokyo 192-8512 Japan; 4 Graduate School of Pharmaceutical Sciences, The University of Tokyo, Tokyo, 113-0033 Japan; 5 Laboratory of Veterinary Pathology, Tokyo University of Agriculture and Technology, Fuchu, Tokyo 183-8509 Japan

## Abstract

Aberrant crypt foci (ACF), the earliest precursor lesion of colorectal cancers (CRCs), are a good surrogate marker for CRC risk stratification and chemoprevention. However, the conventional ACF detection method with dye-spraying by magnifying colonoscopy is labor- and skill-intensive. We sought to identify rat and human ACF using a fluorescent imaging technique that targets a molecule specific for ACF. We found that glutathione S-transferase (GST) P1-1 was overexpressed in ACF tissues in a screening experiment. We then synthesized the fluorogenic probe, DNAT-Me, which is fluorescently quenched but is activated by GSTP1-1. A CRC cell line incubated with DNAT-Me showed strong fluorescence in the cytosol. Fluorescence intensities correlated significantly with GST activities in cancer cell lines. When we sprayed DNAT-Me onto colorectal mucosa excised from azoxymethane-treated rats and surgically resected from CRC patients, ACF with strong fluorescent signals were clearly observed. The ACF number determined by postoperative DNAT-Me imaging was almost identical to that determined by preoperative methylene blue staining. The signal-to-noise ratio for ACF in DNAT-Me images was significantly higher than that in methylene blue staining. Thus, we sensitively visualized ACF on rat and human colorectal mucosa by using a GST-activated fluorogenic probe without dye-spraying and magnifying colonoscopy.

## Introduction

Colorectal cancer (CRC) is one of the leading causes of cancer-related deaths worldwide^1^. Over the past several decades, several attempts have been made to enable the early detection of CRC and adenoma (polyps) including fecal occult blood testing^2^, sigmoidoscopy^3^, fecal DNA tests^4^, capsule endoscopy^5^, and computed tomographic colonography^6^. However, an efficient and satisfactory method and a strategy for the stratification of high-risk groups of CRCs have not yet been established. Moreover, although many studies on CRC chemoprevention have been performed using adenomas (polyps) as target lesions^7–11^, a useful chemopreventive agent has not been established to date.

Aberrant crypt foci (ACF) have been described as minute precancerous lesions consisting of methylene blue stainable, large, and thick crypts in the rodent colorectum. Since ACF are small lesions with a diameter less than approximately 1 mm on colorectal mucosa, they are generally invisible to the naked eye but identifiable only by stereoscopic microscopy after methylene blue staining^12^. We previously succeeded in identifying human ACF *in situ* using magnifying colonoscopy with methylene blue staining, and showed that the number of rectal ACF increased in a stepwise fashion from normal subjects to adenoma patients, and then to cancer patients, suggesting that ACF are precursor lesions to the adenoma–carcinoma sequence in humans^13, 14^. Subsequently, other investigators have shown that the number of rectal ACF in patients with colorectal tumors is significantly higher than in normal subjects^15, 16^. Thus, ACF are an ideal surrogate marker for quantifying CRC risk. Moreover, we and other investigators recently performed chemopreventive studies using ACF instead of adenomas as target lesions, and showed that ACF were eradicated in a short period (in only a few months) by chemopreventive agents^17, 18^, while chemoprevention of adenomas took much longer (at least one or two years). In this context, much attention has been paid to chemoprevention trials in which ACF are used as targets; such trials require the very short-term administration of candidate agents to cancer-free subjects, which results in far fewer adverse effects and higher compliance. However, some investigators have reported that ACF counting by methylene blue staining was not reproducible in their studies^19, 20^. Various reasons have been proposed to account for their results: (1) mucosal staining with methylene blue, including pre- and post-stain washing with water, takes a long time and is very laborious; (2) magnified observations in proximity to colorectal mucosa require a high degree of skill and are time consuming; and, (3) only the rectum was observed for ACF counting in most studies because it is difficult to observe ACF in the entire colorectum. Thus, a new simple technology for ACF detection without dye staining and magnifying colonoscopy is greatly needed.

Molecular imaging is a newly emerging technique whereby the cellular and molecular processes of living cells can be tracked and visualized. Recently, attempts to visualize colorectal polyps as well as CRCs using a fluorescent-labeled antibody or peptide have been reported^21–23^. Moreover, to date, only one report of the molecular detection of ACF using anti-CD44 antibody exists^24^. However, the latter was a very brief report that presented only one image of molecular detection; the efficiency and reproducibility of the method so far remains unclear.

Several investigators have reported the overexpression of various genes in ACF, including c-MET, Cadherin-1, β-catenin, and epidermal growth factor receptor (EGFR), etc^25–28^. We have reported that glutathione S-transferase (GST) P1-1, a phase II detoxifying enzyme, is highly expressed in human ACF tissue^29, 30^. The aforementioned genes are possible candidates for the molecular imaging of ACF. Therefore, in this study, we first investigated the expression of these candidate genes in ACF to establish a new molecular imaging system. We then selected the GSTP1 gene as a target and, using our original GST-activated fluorogenic probe, we detected ACF in a rodent model and in surgically excised human colorectal tissues.

## Results

### Gene mRNA expression levels in human ACF tissues

**Figure 1:**
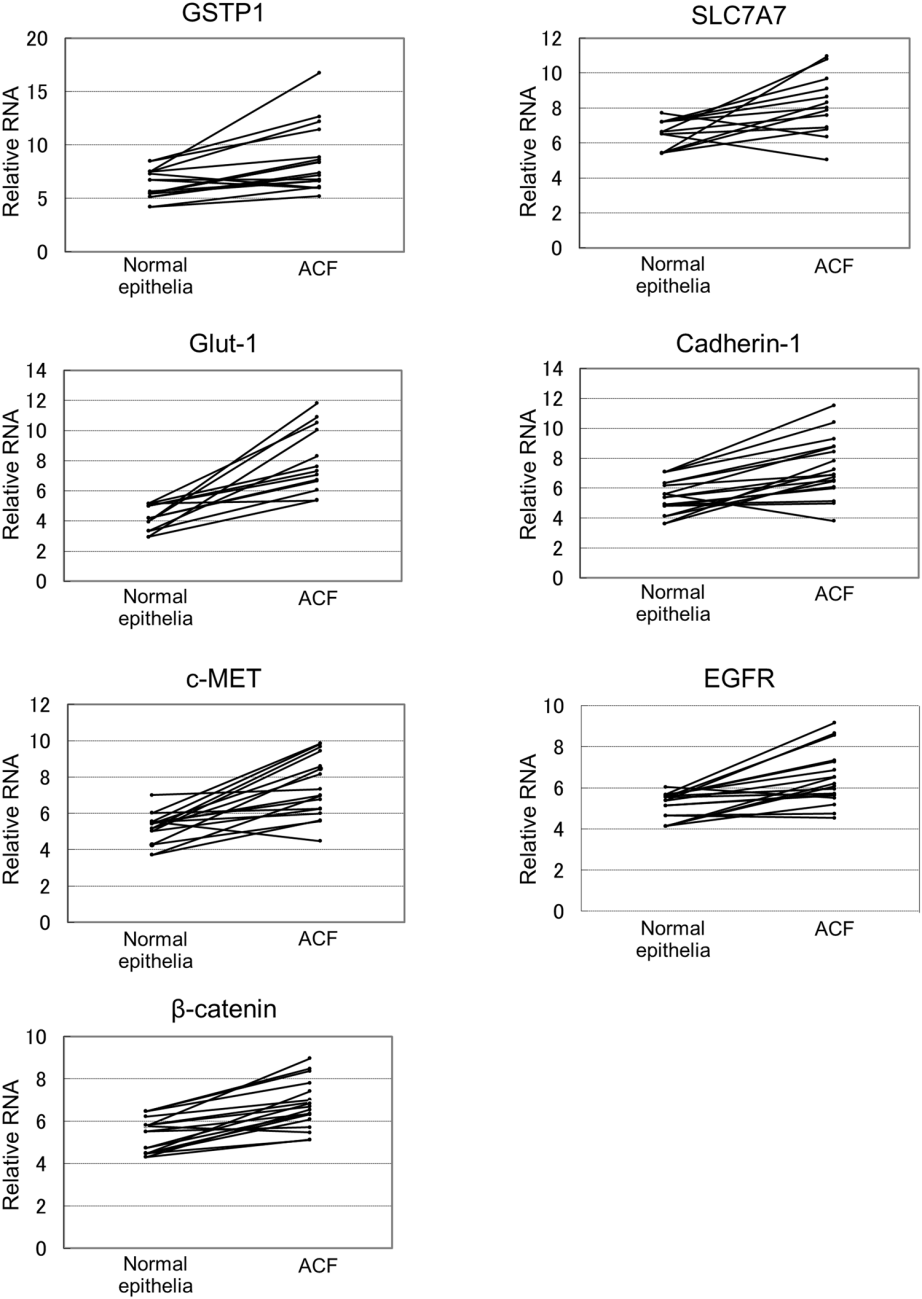
Gene mRNA levels in human ACF tissues. ACF and normal colorectal tissues were biopsied under magnifying colonoscopy. Total RNA was extracted and reverse transcribed, and a TaqMan assay was performed to quantitate mRNA levels for each gene. The 18 S gene was used as an internal control.

We first measured mRNA levels for the 20 candidate genes that had been reported to be highly expressed in colorectal ACF and/or tumors in rats and humans (Fig. 1). Levels of mRNA for GSTP1, Glut-1, c-MET, β-catenin, SLC7A7, Cadherin-1, and EGFR in ACF tissues were significantly (2.0–3.0-fold) higher than those in surrounding normal epithelia (*p* < 0.05). On the other hand, there were no significant differences in mRNA levels for Fzd1, iNOS (NOS2), CD44, COX-2, c-KIT, EPHB3, CD24, Glut-4 (SLC2A4), ADAM17, PTGER2, CDK4, cathepsin B1, and GPX-2 between ACF and the surrounding normal epithelia (Supplementary Fig. 1).

Immunohistochemical analysis of protein expression for these genes revealed that Glut-1 and GSTP1-1 stained strongly in human ACF tissues, whereas the remaining proteins were stained relatively weakly compared with surrounding normal epithelia (Supplementary Fig. 2). The high expression of GSTP1-1 in human ACF tissue was consistent with our previous reports^29, 30^. We also confirmed that Glut-1 and GSTP1-1 were highly expressed in ACF from azoxymethane (AOM)-treated rats (Supplementary Fig. 3). Based on these results, we concluded that GSTP1-1 and Glut-1 would be the most appropriate molecular targets, and therefore tried to detect ACF using a GSTP-activated fluorogenic probe (DNAT-Me) or a fluorescent D-glucose analogue (2-NBDG) as a probe for Glut-1^31^. However, as described in the Discussion section, since imaging with 2-NBDG was inferior to that with DNAT-Me, we reported herein the results for DNAT-Me only.

### Confocal microscopic imaging of living cells using DNAT-Me

**Figure 2:**
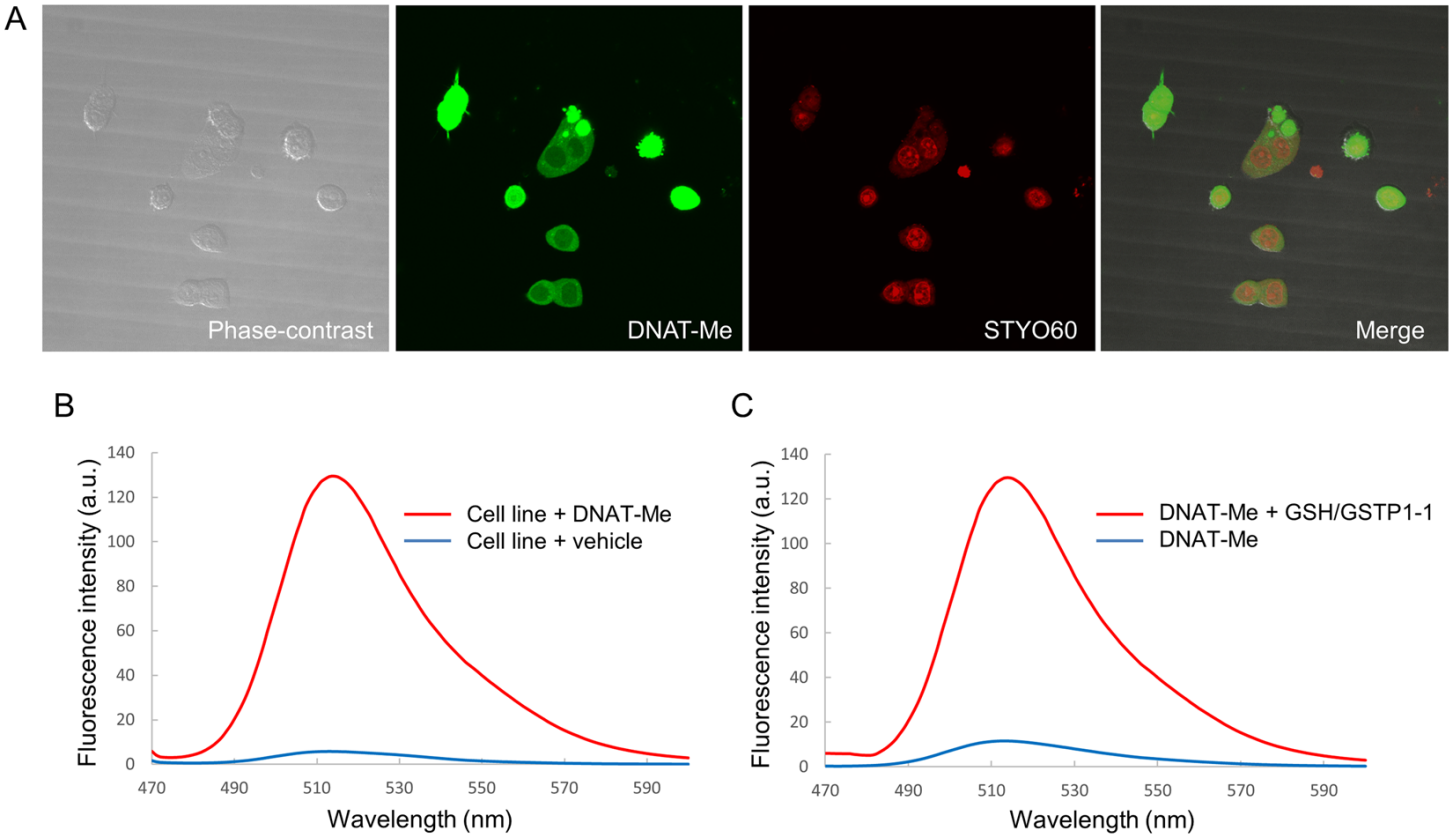
Confocal microscopic imaging of live cells using DNAT-Me. (**A**) M7609 cells were cultured overnight on chamber slides, incubated with 10 μM DNAT-Me and 1 μM SYTO60 for 15 min, and observed under phase-contrast and confocal microscopes. (**B**) Fluorescence emission spectra of live cells incubated with DNAT-Me were analyzed spectrophotometrically from 470–600 nm, with excitation at 488 nm. (**C**) Similarly, fluorescence emission spectra of DNAT-Me reacted with glutathione S-transferase P1-1 and glutathione were analyzed from 470–600 nm with excitation at 488 nm.

Since cell lines have not been established from ACF tissues, we first investigated whether a colon cancer cell line (M7609) with appreciable GSTP1-1 activity could provide a clear fluorescence image following incubation with DNAT-Me and imaging using a confocal fluorescence microscope (Fig. 2). Live M7609 cells were observed as round or oval shapes, with several projections, under phase-contrast microscopy. After incubation with DNAT-Me and the nucleic acid-specific stain SYTO60, bright green fluorescence was clearly observed predominately in the cytoplasm of each cell, while the nucleus exhibited bright red fluorescence (Fig. 2A). Fluorescence emission spectra obtained from live cells showed that the maximum wavelength of emission was 516 nm (Fig. 2B). This was consistent with the fluorescence spectra obtained from the fluorophore released from DNAT-Me reacted with GSTP1-1 (Fig. 2C), and is also consistent with the expected spectra from the structure of DNAT-Me^32^.

### Correlation between fluorescence intensity and GST activity in various cell lines

**Figure 3:**
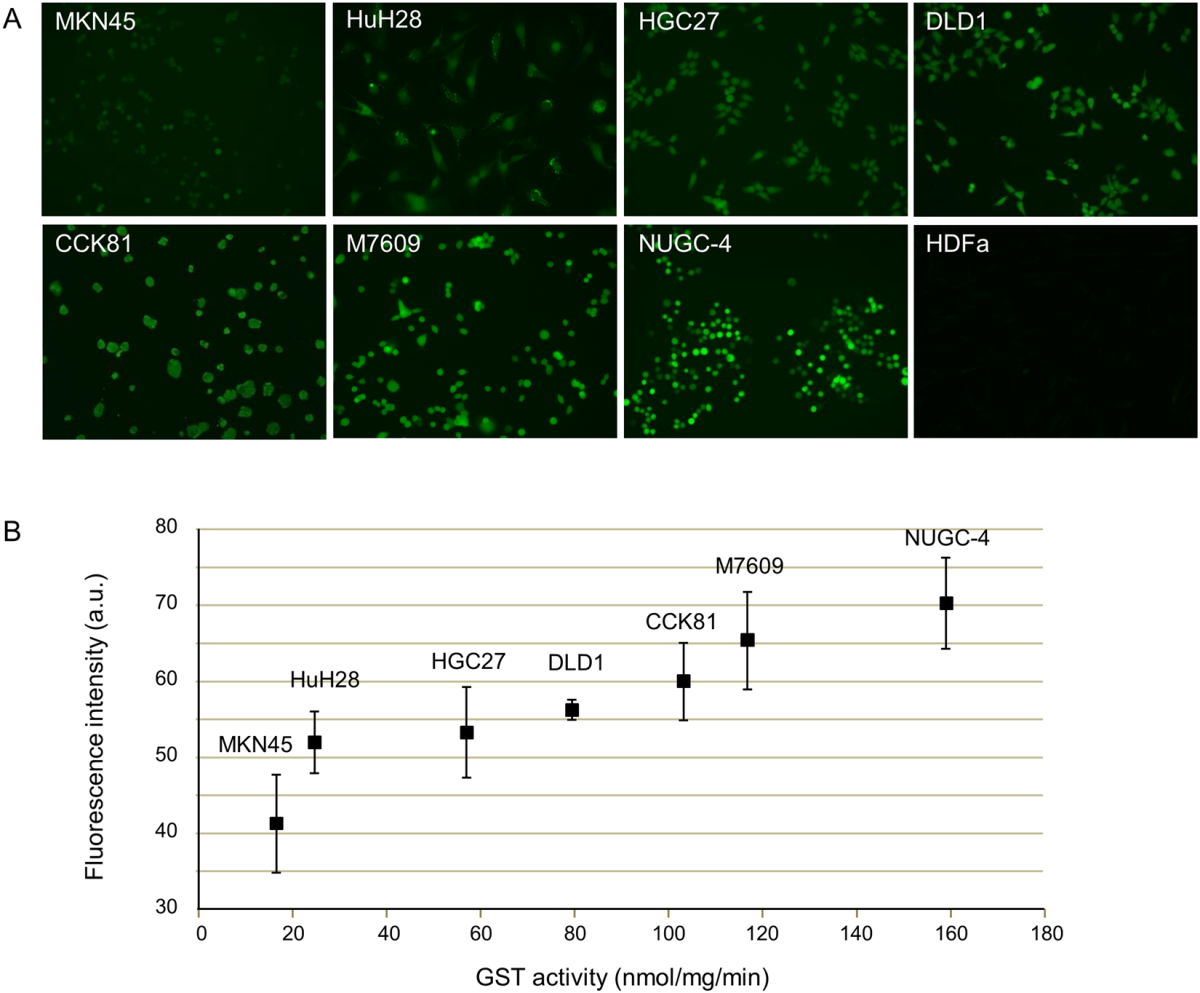
Correlation between GST activity and fluorescence intensity in various cancer cell lines. (**A**) Seven cancer cell lines (MKN45, HuH-28, HGC27, DLD1, CCK81, M7609, and NUGC-4) were incubated with DNAT-Me at 10 μM for 10 min and observed under fluorescence microscopy with excitation at 488 nm. Fluorescence intensity per cell in each cell line was quantitated using software provided by the manufacturer, as described in Materials and Methods. A primary normal adult human dermal fibroblast line (HDFa) was used as a negative control. (**B**) The cytosolic fraction was extracted from each cell line, and GST activity was measured using 1-chloro-2,4- dinitrobenzene and glutathione as substrates.

We next analyzed the correlation between the fluorescence intensity of DNAT-Me and GST activity in seven cancer cell lines. MKN45, HuH-28, HGC27, DLD1, CCK81, M7609 and NUGC-4 cell lines were incubated with DNAT-Me and observed by fluorescence microscopy (Fig. 3A). The respective fluorescence intensities were 41.3 ± 6.4, 52.0 ± 4.0, 53.3 ± 6.1, 56.3 ± 1.2, 60.0 ± 5.3, 65.3 ± 6.4, and 70.3 ± 6.1 AU/cell. The GST activities of these cell lines were 16 ± 2.0, 22 ± 3.1, 57 ± 4.3, 79 ± 4.6, 104 ± 15, 117 ± 13, and 158 ± 21 nmol/mg/min, respectively. In primary normal fibroblasts (HDFa), which have very low GST activity (<15 nmol/mg/min) and were used as a negative control, the fluorescence intensity was negligible (3.2 ± 0.6 AU/cell). There was a significant correlation between fluorescence intensity and GST activity in the cancer cell lines (ρ = 0.95, *p* < 0.01; Fig. 3B).

### *Ex vivo* molecular imaging of ACF in AOM-treated rats

**Figure 4:**
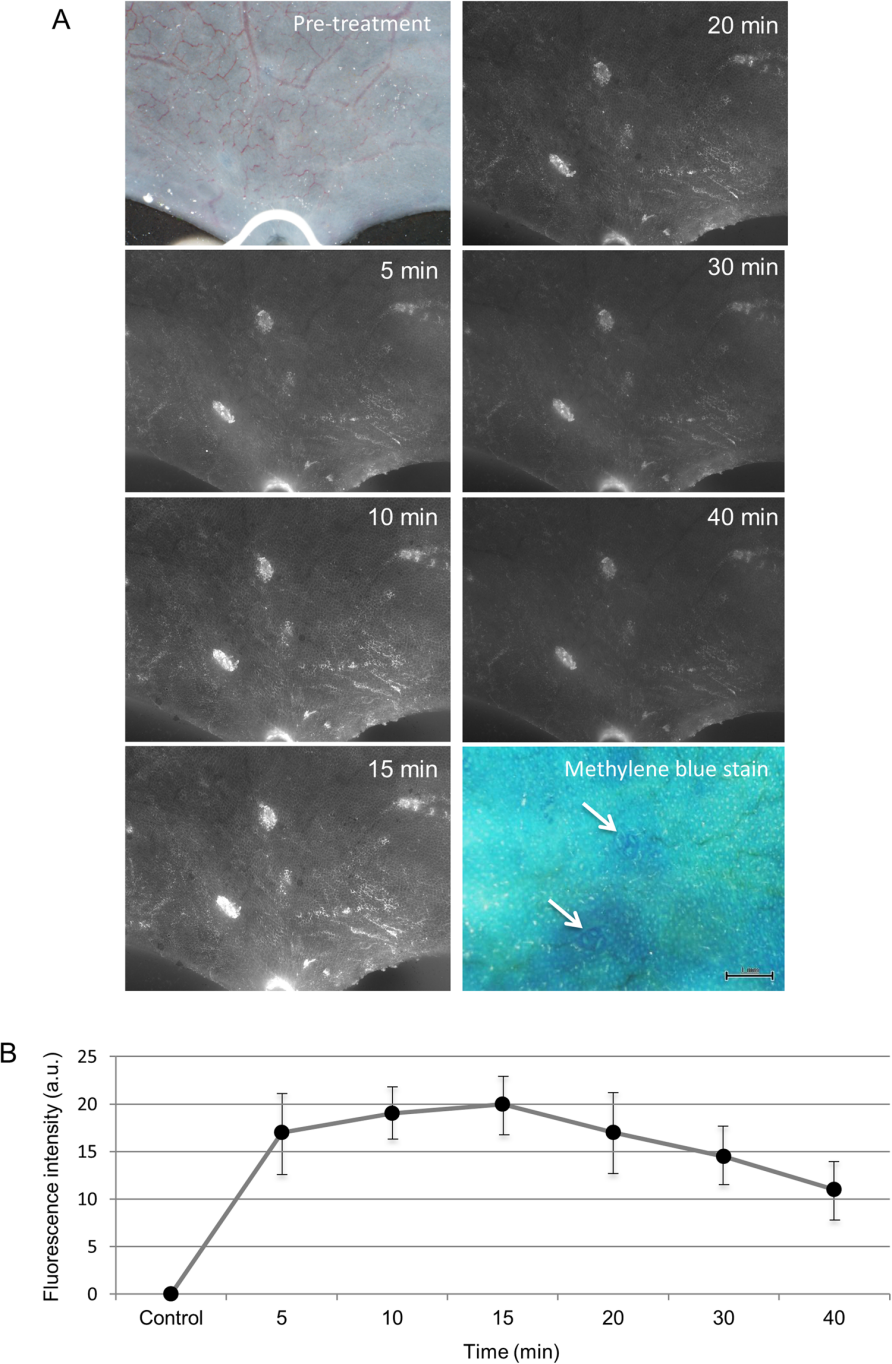
*Ex vivo* molecular imaging of ACF in azoxymethane (AOM)-treated rats. (**A**) The colorectum was excised from an AOM-treated rat, placed mucosal side up, washed and sprayed with 10 mL of DNAT-Me (200 μM). The colorectum was observed using a fluorescence stereomicroscope before reaction (white light) and at 5, 10, 15, 20, 30, and 40 min after reaction. Specimens were then fixed in formalin and the mucosal surface was stained with methylene blue, after which ACF were observed. (**B**) The fluorescence intensity of ACF at each time point was quantitated, and a chronological curve generated using the mean ( ± SD) fluorescence intensity at each time point from five ACF.

We observed ACF in the colorectums of five AOM-treated rats *ex vivo* by fluorescence imaging with DNAT-Me, and evaluated chronological changes in the images. Representative chronological images are shown in Fig. 4. After the topical application of DNAT-Me onto excised rat colorectal mucosa, ACF started to show a weak signal within a few minutes and were well visualized at 5 min. The signal intensity increased, reaching a plateau at 15 min and then gradually decreased (Fig. 4A). We then sprayed methylene blue onto the colon and observed ACF using this conventional ACF detection method. We were able to visualize two ACF consisting of two and three crypts, respectively, in their corresponding locations. Fluorescence images were much clearer than conventional methylene blue images of ACF, although both ACF were very small with only a few crypts. We obtained similar results in experiments with the remaining four rats. We measured fluorescence intensity over time in five ACF randomly selected from each rat and used the data to generate a mean chronological curve of fluorescence intensity in ACF (Fig. 4B). These results demonstrated that ACF could be clearly visualized following the application of DNAT-Me in AOM-treated rats and that 15 min was sufficient time for the optical visualization of ACF.

### *Ex vivo* molecular imaging of ACF in human resected colorectum

**Figure 5:**
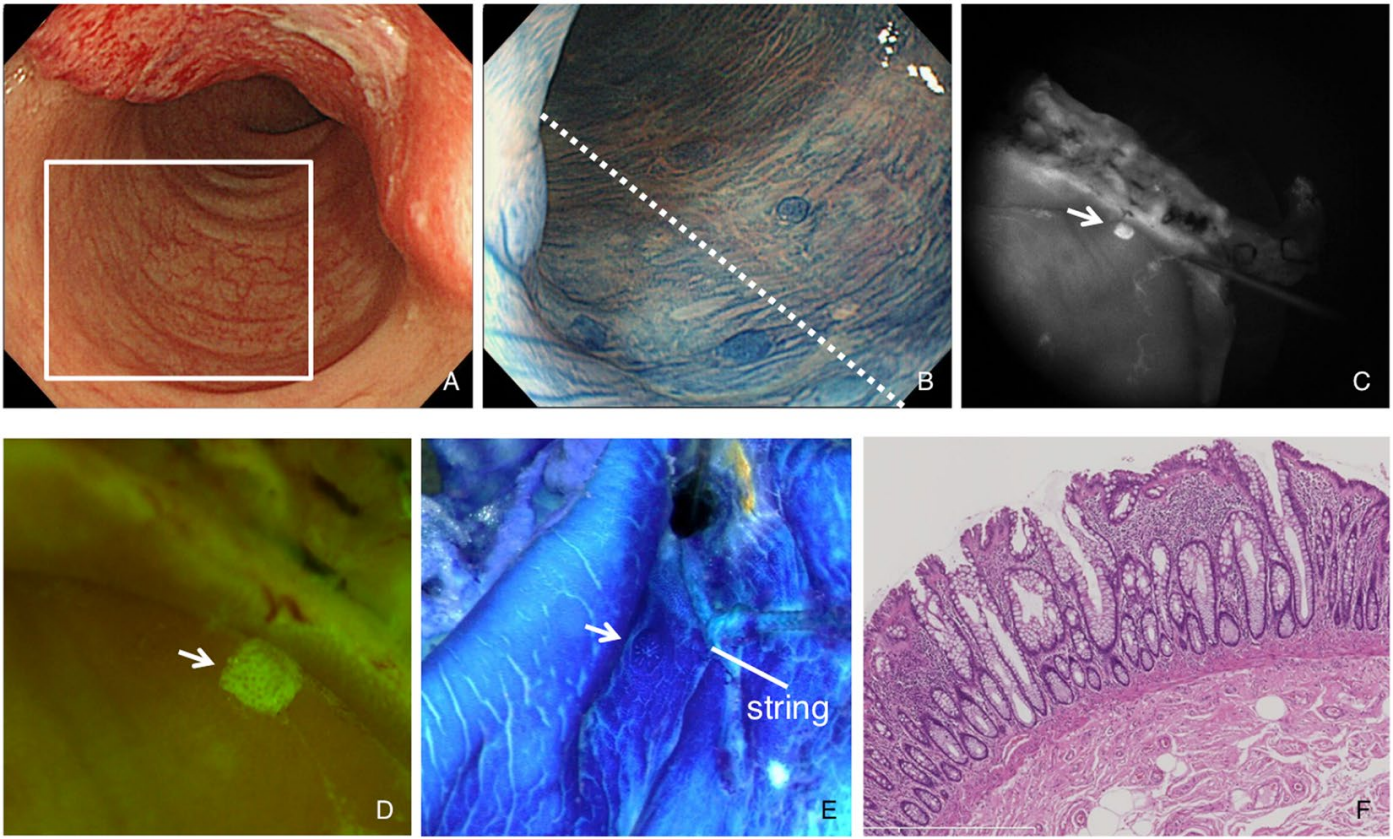
*Ex vivo* molecular imaging of ACF in human resected colorectum. A colonoscopy was performed in a patient with colorectal cancer (CRC) by using a high-definition magnification colonoscope. (**A**) White light finding for CRC. The squared area corresponds to Panel B. (**B**) ACF identified by methylene blue staining with magnifying observation. After surgical resection, the colon was cut along the dotted line. (**C**) An ACF identified in surgically resected colon after DNAT-Me application with fluorescence imaging (arrow). (**D**) A pseudocolored image of the ACF in Panel C (arrow). (**E**) An ACF observed in formalin-fixed colon observed under a stereomicroscope after methylene blue staining (arrow). Mucosa adjacent to the ACF was marked with a string suture. (**F**) Histological findings for the ACF.

Based on the results of animal experiments, we undertook the molecular detection of human ACF by applying DNAT-Me on colorectal mucosa surgically resected from seven patients with CRCs, and compared results with those from methylene blue staining. Representative images of ACF surrounding CRC identified by methylene blue staining under magnifying colonoscopy in a pre-operative observation of case 1 are shown in Fig. 5A and B. Sigmoid colon tissue, including the cancer lesion, was surgically removed, cut along the dotted line, and opened (Fig. 5B). After DNAT-Me treatment, a strong fluorescence signal was detected in the location corresponding to where ACF had been observed in a pre-operative examination (Fig. 5C,D). The mucosa adjacent to the ACF was marked with a string suture. The formalin-fixed tissue was then stained again with methylene blue, and the existence of ACF, densely stained with methylene blue, was confirmed adjacent to the string under a stereomicroscope (Fig. 5E). Histological examination of the ACF lesions revealed enlargement and branching of the crypts, characteristic of the histological findings of ACF (Fig. 5F). The location map of eight ACF with DNAT-Me treatment was highly compatible with that found after methylene blue staining. Similarly, in cases 2 and 3, we also marked ACF regions with string suture after DNAT-Me imaging, and confirmed that fluorescence signals were indeed from ACF lesions. We were able to clearly detect ACF with DNAT-Me treatment in all of the seven cases using the fluorescence imaging system.

**Figure 6:**
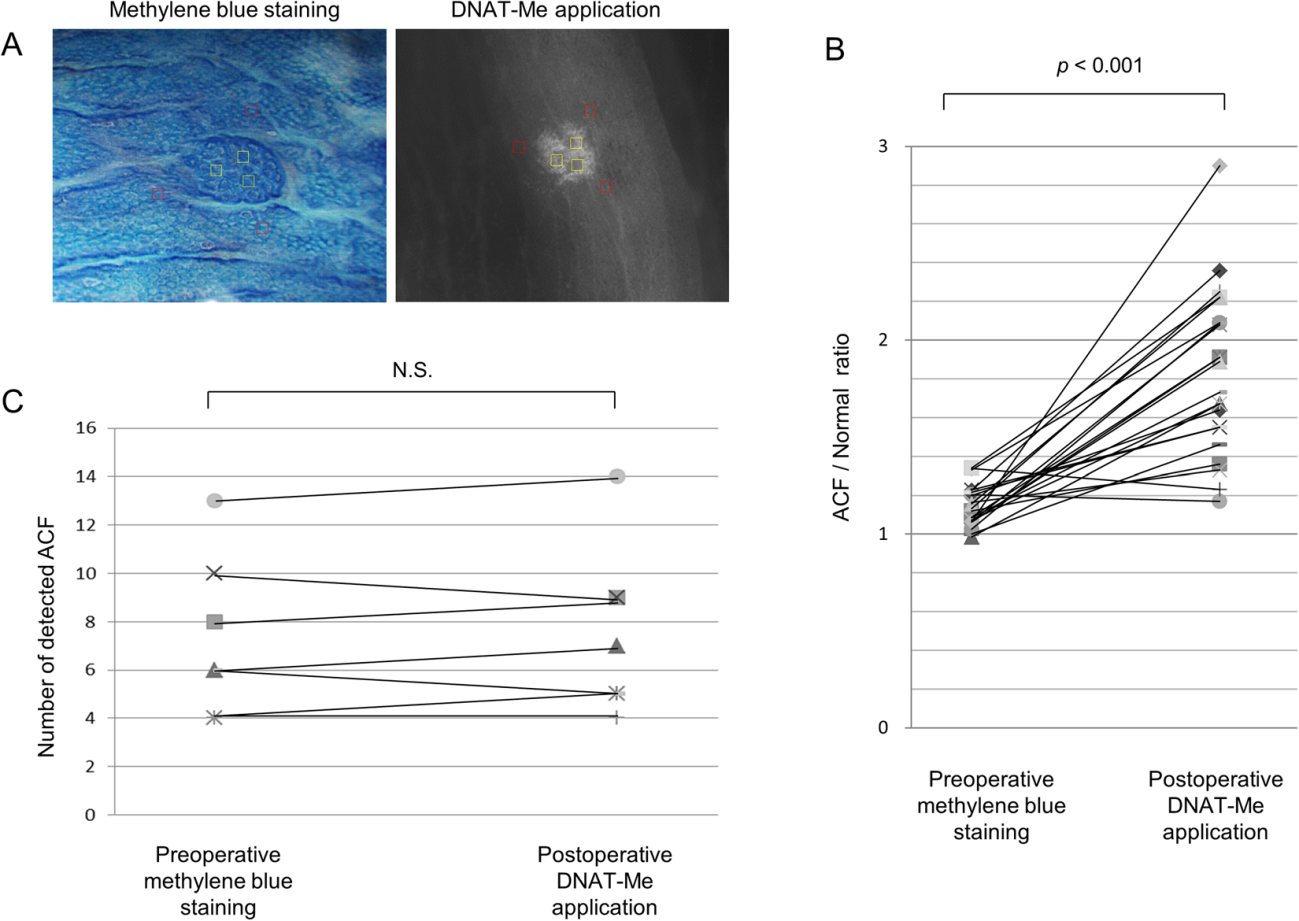
Comparison of signal-to-noise (S/N) ratios for ACF, and ACF counts with methylene blue staining and DNAT-Me imaging. (**A**) Representative images of an ACF obtained with methylene blue staining or DNAT-Me application. Three regions of interest (ROIs) of 250 μm × 250 μm (yellow squares) in the ACF and three ROIs (red squares) in the background were randomly set, and the S/N ratio calculated using image processing software. (**B**) Comparison of S/N ratios for ACF between methylene blue staining and DNAT-Me imaging. The average S/N ratios in each of the 23 ACF in pre- and postoperative examinations were plotted. (**C**) A comparison of ACF numbers as determined by methylene blue staining and DNAT-Me imaging. The number of ACFs in the 7**-**cm area distal (or proximal) to the cancer was counted by preoperative methylene blue staining and by postoperative DNAT-Me application. N.S., not significant.

A total of 56 ACF were identified in seven patients by DNAT-Me molecular imaging. The signal-to-noise (S/N) ratios in images obtained after DNAT-Me application and methylene blue staining were calculated based on the signal intensities of ACF and the surrounding normal mucosa (Fig. 6A). The mean S/N ratio for 23 randomly selected ACF in DNAT-Me images was 1.82 ± 0.42, which was significantly higher than that for images obtained after methylene blue staining (1.15 ± 0.10; (*p* < 0.01; Fig. 6B). This suggests that the molecular detection of ACF by DNAT-Me is more sensitive than the conventional methylene blue stain method. The number of ACF determined by postoperative DNAT-Me imaging was virtually identical (within ±1) to that determined by preoperative methylene blue staining in all seven patients (Fig. 6C). Thus, the molecular detection of ACF by DNAT-Me was appreciably sensitive, and appeared to be superior to the conventional methylene blue method in that it was much simpler, faster, and had a higher S/N ratio than the conventional method.

## Discussion

In this study, we clearly visualized ACF using our original GST-activated fluorogenic probe (DNAT-Me) in normal appearing mucosa around cancerous lesions of colorectum resected from patients with CRCs, as well as in colorectal mucosa of AOM-treated mice. This is essentially the first study to identify ACF in human colorectum as well as in animals using molecular imaging technology rather than methylene blue staining. Moreover, our method does not require laborious washing after DNAT-Me application because it is fluorescently quenched and transparent, thereby resulting in very low background noise. Therefore, the S/N ratio in images obtained using DNAT-Me was higher compared with those obtained using methylene blue staining, indicating the high sensitivity of this technology. In fact, many small ACF that consisted of only a few crypts could be clearly identified by this method, as described in Fig. 4. In addition, our method does not require high magnification to observe ACF because the fluorescence intensity is strong and readily identifiable (Figs 4 and 5). These properties make it much easier to identify ACF at low magnification, and thereby enables the observation of ACF not only in part of the rectum, but also in the entire colorectum. Thus, our method of ACF observation is very simple and highly sensitive, potentially facilitating the diagnosis of a high-risk patient group and the evaluation of chemopreventive agents for CRCs.

Most fluorescent probes previously developed for molecular imaging are inherently fluorescent, and therefore require extensive washing to minimize background noise. However, DNAT-Me, which we designed and synthesized, is fluorescently quenched and activated only by GST inside cells^32^. When the NO_2_ group of DNAT-Me is enzymatically substituted with glutathione (glutathionylation) in cells, it exhibits very strong fluorescence activity, and therefore does not require extensive washing after topical spraying. We speculate that these fluorogenic characteristics were responsible for the clear fluorescent ACF images obtained showing a high S/N ratio, in sharp contrast to the adjacent normal mucosa (Fig. 5). We found in the first part of this study that Glut-1 was also overexpressed in human ACF (Fig. 1). Recently, the fluorescent probe 2-NBDG, a substrate for Glut-1, has been developed^31^. We used 2-NBDG for the molecular imaging of ACF on colorectal mucosa resected from rats and humans. However, it produced ACF images of inferior quality relative to those for DNAT-Me, with a lower S/N ratio and higher background noise in both rat and human colorectal mucosa. This may be partly explained by the fact that 2-NBDG is fluorescently active but not quenched. Although it is more difficult to develop a fluorogenic probe that can be activated by the membrane transporter, Glut-1, we are now trying to generate such a new fluorogenic Glut-1-targeting probe.

In our present study using a rat model, the fluorescent signal intensity in ACF was enhanced in a time-dependent manner, reaching a plateau at 15 minutes. Similarly, ACF could be clearly identified in images of human colorectal mucosa within a reaction time of 15 min. However, enzyme activity in excised tissues generally decreases with time. Therefore, it is expected that the reaction time of DNAT-Me would be shorter with *in vivo* use. In fact, in our *in vivo* experiment in rats, we were able to clearly detect ACF 10 min after DNAT-Me administration; the fluorescence intensity of ACF gradually decreased after 20 min and almost disappeared at 30 min (Supplementary Fig. 4). The optimal reaction time for the *in vivo* use of DNAT-Me should be investigated in detail in future studies. Moreover, we are currently evaluating the safety of DNAT-Me in animals in order to detect ACF in humans using high-resolution fluorescence endoscopy. With respect to clinical applications, two routes exist for the administration of probes: topical administration by direct spraying on the colorectum and intravenous administration. Our data suggest that the topical administration of DNAT-Me has an appreciably high sensitivity for the detection of ACF. However, it will also be important to evaluate the utility of our probe when administered intravenously.

Cytosolic GSTs comprise six isozymes (α, μ, π, θ, ω, and ζ)^33^. DNAT-Me was synthesized as a membrane-permeable derivative from DNAF1 that specifically binds to a π class GST (GSTP1-1) with a very high *k*_cat_/*K*_M_ value^32^. Moreover, it has been reported that GST activity reflects GSTP1-1 expression in colorectal neoplasms^34^. In this context, it appears that fluorescence intensity of the images in this study reflected GSTP1-1 expression in ACF. In addition, we have found that GSTP1-1 plays a pivotal role in colorectal carcinogenesis via its anti-apoptotic effect^29, 30^. Therefore, it is a reasonable strategy to target GSTP1-1 for the molecular imaging of ACF as precancerous lesions.

The conventional method for ACF identification involving methylene blue staining is based on the difference in absorption of methylene blue between ACF cells and surrounding normal epithelial cells. Since methylene blue is absorbed into normal epithelial cells as well as into ACF cells, the background signal is very high and the S/N ratio is low. Moreover, washing out residual methylene blue is time-consuming and laborious. In contrast, our method is based on the difference in GSTP1-1 expression between ACF cells and normal epithelial cells: Normal colorectal epithelial cells express a very low level of GSTP1-1; however, GSTP1-1 is expressed in ACF, adenomas, and subsequent CRCs. Since our probe is transparent and is not activated in the absence of GST activity, it yields a higher S/N ratio with lower background signal as compared with methylene blue staining (Fig. 6). In fact, we found that ACF, adenomas, and subsequent cancer lesions were clearly detectable using DNAT-Me and the fluorescent imaging system (Supplementary Fig. 5). Moreover, the number of ACF in samples treated with DNAT-Me postoperatively was almost identical to that with preoperative methylene blue staining. However, the number of colorectal tissues examined in this study was limited. Therefore, the sensitivity and specificity of DNAT-Me should be prospectively compared with that of methylene blue in a large-scale clinical trial.

GSTP1-1 is overexpressed in various cancers, including gastric cancer, lung cancer, ovarian cancer, and precancerous lesions^35–38^. Therefore, our data suggest that a fluorogenic probe targeting GSTP1-1 for molecular imaging would also be very useful for the early diagnosis and/or evaluation of chemopreventive agents for these types of cancers.

In conclusion, we clearly detected ACF on colorectal mucosa specimens surgically resected from patients with CRCs, and in specimens excised from AOM-treated rats using a GST-activated fluorogenic probe with a fluorescence imaging system. This new molecular imaging technique targeting GSTP1-1 does not require laborious washing with water because it is transparent and fluorescently quenched. Moreover, it displays superior sensitivity with a higher S/N ratio as compared with conventional methylene blue staining, and does not require magnifying colonoscopy. This technology, combined with colonoscopy, may enable the easy and sensitive detection of ACF as a surrogate marker for risk stratification and the evaluation of chemopreventive agents for CRCs.

## Methods

### Study design

In the first step of this study, we investigated the mRNA expression of 20 genes in ACF tissues as targets for molecular imaging: GSTP1, glucose transporter-1 (Glut-1), c-MET, β-catenin, SLC7A7, Cadherin-1, EGFR, Fzd1, inducible nitric oxide synthase (iNOS, NOS2), CD44, cyclooxygenase-2 (COX-2), c-KIT, EPHB3, CD24, Glut-4 (SLC2A4), ADAM17, prostaglandin E receptor 2 (PTGER2), cyclin dependent kinase 4 (CDK4), cathepsin B1, and glutathione peroxidase-2 (GPX-2). These genes were previously reported to be highly expressed in colorectal ACF and/or tumors in rats and humans. We ultimately selected GSTP1-1 as the target molecule, and in the next step, performed *in vitro* imaging experiments of cultured cell lines using our original GST-activated fluorogenic probe; we also detected ACF in a rat model of colorectal carcinogenesis and in human colorectum surgical specimens. This study was approved by the institutional review board of Tokushima University Hospital (Tokushima, Japan). All human samples were obtained from patients who provided written informed consent before endoscopy and surgery. All methods were performed in accordance with the relevant guidelines and regulations.

### Human ACF tissue

Human ACF tissues for gene expression analysis were obtained by biopsy from 12 patients with colorectal adenoma and two patients with CRC. The average age of the patients was 67.3 ± 10.4 years and the male-to-female ratio was 6:8. Total colonoscopy was performed by high-definition magnification colonoscopy (CF-H260AZI, Olympus Corp., Tokyo, Japan), and ACF in the rectosigmoidal colon were then observed as described previously^13, 14, 39^. A biopsy of ACF tissue was taken, put into RNA later^®^ RNA stabilization reagent (Life Technologies, Inc., Rockville, MD) and unfolded on a rubber plate; the central part of the ACF tissue was then punched out with a biopsy stick (1.0 mm diameter) under a stereomicroscope, and RNA extraction was performed. For histological analysis, a biopsy of ACF tissue was obtained in the same manner and fixed in 10% neutral buffered formalin.

ACF were defined as lesions in which crypts were more darkly stained with methylene blue than normal crypts and had larger diameters, often with oval or slit-like lumens and thicker epithelial linings^12, 13^.

### Real-time PCR

Quantitative real-time PCR (TaqMan assay) was performed as described previously^29^. In brief, total RNA was extracted using an RNeasy Mini kit (QIAGEN, Hilden, Germany) and was reverse transcribed into complementary DNA (cDNA). The probe and primer sets for each gene from the TaqMan gene expression assay (Applied Biosystems, Foster City, CA) are described in Supplementary Methods. Quantitative PCR was performed using a StepOnePlus Real Time-PCR System (Applied Biosystems). PCR amplification conditions were one cycle at 50 °C for 2 min and 95 °C for 10 min, followed by 40 cycles at 95 °C for 15 s and 60 °C for 1 min. The measured value was calculated using a comparative threshold-cycle number (Ct) method, and the*18S* gene was used as an internal control. In order to determine the efficiency of each Taqman gene expression assay, standard curves were generated by serial dilution of cDNA, and quantitative evaluation of target and internal control gene levels was performed by measuring Ct.

### Cell lines

The human CRC cell lines, CCK-81 and DLD-1, and the gastric cancer cell line, MKN45, were obtained from the Health Science Research Resources Bank (Osaka, Japan). The human gastric cancer cell lines, HGC-27 and NUGC-4, and the human cholangiocellular cancer cell line, HuH-28, were obtained from the RIKEN Bio Resource Center (Ibaraki, Japan). The human CRC cell line, M7609, was kindly provided by Dr. S Machida (Hirosaki University, Hirosaki, Japan). Adult human dermal fibroblasts (HDFa), as a primary normal adult human dermal fibroblast cell line from skin, were purchased from Thermo Fisher Scientific Inc. (Wilmington, DE). Each cell line was cultured in the recommended medium containing 10% fetal calf serum at 37 °C with 5% CO_2_.

### Fluorescent probe

A GST-activated fluorogenic probe DNAT-Me (molecular weight 510; Supplementary Fig. 6) was synthesized as previously described^32^. DNAT-Me was dissolved in DMSO at a concentration of 2 mM and stored at −20 °C.

### Measurement of GSTP1-1 activity

Each cell line was harvested using a cell scraper, incubated in hypotonic buffer (pH 7.4; 10 mM Tris, 1.5 mM MgCl_2_) for 20 min at 4 °C, homogenized using a Dounce homogenizer, and centrifuged at 10,000 × *g* for 30 min at 4 °C to collect cytosolic proteins. GSTP1-1 activity was measured using 1-chloro-2, 4-dinitrobenzene (CDNB) as a substrate, as described previously^40^. In brief, protein samples (10–50 μL) were added to 1 mL of 0.1 M sodium phosphate buffer (pH 6.5) containing 1.3 mM CDNB and 2.5 mM reduced glutathione (Sigma-Aldrich), and the absorbance at 343 nm was measured at 25 °C.

### *In vitro* molecular imaging of cancer cell line

M7609 cells cultured in chamber slides were incubated with 10 μM DNAT-Me (0.5% DMSO in PBS) and 1 μM SYTO60 for nuclear counterstaining without fixation for 15 min. They were washed with PBS twice, and observed by confocal microscopy (A1 system, Nikon, Tokyo, Japan) with excitation at 488 and 652 nm, respectively. Fluorescence emission spectra of cells incubated with DNAT-Me were measured spectrophotometrically from 470 to 600 nm with excitation at 488 nm. A recombinant GSTP1-1 and glutathione were added to a DNAT-Me (200 μM) solution in a quartz cuvette, and fluorescence emission spectra were measured from 470 to 600 nm with excitation at 488 nm.

For the evaluation of fluorescence intensities, 1 × 10^5^ cells seeded on 35-mm dishes were incubated with DNAT-Me (10 μM) for 10 min, and observed by fluorescence microscopy with excitation at 488 nm (BZ 9000, Keyence Corp. Osaka, Japan). The fluorescence intensity of individual cells in each cell line was quantitated using software provided by the manufacturer. At least 150 cells were quantitated for each cell line and each experiment was repeated three times.

### *Ex vivo* molecular imaging of ACF in a rat model

AOM was administered subcutaneously to five male F344 rats, as described previously^41^. They were sacrificed at eight weeks, and entire colorectums were carefully removed and opened longitudinally from the cecum to the anus. Colorectums were washed with warm PBS to remove feces and mucus from the surface of mucosa, onto which 10 mL of DNAT-Me (200 μM) was then sprayed. Chronological fluorescence images were taken and stored digitally using a Macro Zoom Microscope (MVX/DP80, Olympus Corp.). Subsequently, the tissues were fixed in 10% formalin for 24 h, and stained with 0.2% methylene blue to observe ACF under a stereomicroscope. The fluorescence intensity of ACF was quantitated using Image J software and normalized to control levels.

### Fluorescence imaging system

We developed a prototype fluorescence imaging system for the observation of colorectal mucosa by modifying a conventional endoscopy system with a light source CLV-180 (Olympus Corp.; Supplementary Fig. 7). An excitation filter with a transmission wavelength of 465–490 nm was placed between the xenon lamp and the rigid scope, and a barrier filter with a transmission wavelength of 510–550 nm was placed between the scope and the two recording cameras. Fluorescent signals detected using the rigid scope equipped with a prism were sent split to both the fluorescence camera and color camera. The distance between the tip of the rigid scope and the subject was fixed at 50 mm. This system provided a 2.5-fold magnification on a 19-inch monitor screen.

To calculate the S/N ratio, we randomly selected three regions of interest (ROIs) with an area of 250 μm × 250 μm in each ACF image and the adjacent background mucosa, captured all gray-scale or blue-channel grades of intensity, analyzed average intensity in each ROI, and calculated the S/N ratio using WinROOF image processing software (Mitani Corp., Tokyo, Japan) as described previously^42^.

### *Ex vivo* molecular imaging of ACF in human resected specimens

Seven patients with advanced CRC were enrolled. They had already been diagnosed with CRC based on biopsy under colonoscopy and had been scheduled to undergo surgery. Patient characteristics are described in Supplementary Table. High-definition magnification colonoscopy (CF-H260AZI, Olympus Corp.) was used throughout the examination. After observation of the CRC, a 7-cm area distal (or proximal) to the cancer was washed thoroughly with water, stained with 0.2% methylene blue, and washed again with water for the identification of ACF. ACF were counted and numbered under magnification as described previously^13, 14, 39^. All procedures were recorded on videotape and evaluated by two independent expert endoscopists (N.M. and K.O.) trained in ACF observations.

Immediately following resection, colorectal tissues including cancer were immersed in cold PBS and transferred in plastic containers to an imaging room. The samples were washed once with warm PBS to remove mucus on the mucosal surface. Subsequently, the target area was treated with 20 mL of DNAT-Me (200 μM). Tissues were then incubated in warm water at 37 °C for 15 min. Next, samples were washed with PBS and observed using our fluorescence imaging system, as shown in Supplementary Fig. 7, and lesions that showed a strong fluorescence signal compared with the background mucosa were counted and numbered. Some ACF were marked nearby with a string suture. Samples were then fixed in 10% formalin and stained with methylene blue, and ACF were counted and numbered within the next few days.

### Statistics

The mRNA levels for each gene in ACF and surrounding normal epithelia were compared by Wilcoxon signed rank test. The correlation between the fluorescence intensity and GST activity in each cancer cell line was assessed using Pearson’s correlation test. The S/N ratios in images of ACF from methylene blue staining and DNAT-Me application were compared by Wilcoxon signed rank test. Differences were considered significant at *p* < 0.05.

## Electronic supplementary material

Supplementary information
