# Supplementary material for: Molecular imaging of aberrant crypt foci in the human colon targeting glutathione S-transferase P1-1
Source: Sci Rep. 2017 Jul 26;7:6536. doi: 10.1038/s41598-017-06857-x (PMC5529364; doi:10.1038/s41598-017-06857-x)
Supplement: Supplementary file 1 — Supplementary information [file 41598_2017_6857_MOESM1_ESM.pdf]

Supplementary Information

Molecular imaging of aberrant crypt foci in the human colon targeting glutathione S-transferase P1-1

Naoki Muguruma[1], Koichi Okamoto[1], Tadahiko Nakagawa[1], Katsutaka Sannomiya[1], Shota Fujimoto[1], Yasuhiro Mitsui[1], Tetsuo Kimura[1], Hiroshi Miyamoto[1], Jun Higashijima[2], Mitsuo Shimada[2], Yoko Horino[3], Shinya Matsumoto[3], Kenjiro Hanaoka[4], Tetsuo Nagano[4], Makoto Shibutani[5], Tetsuji Takayama[1,*]

[1]Department of Gastroenterology and Oncology, Institute of Biomedical Sciences, Tokushima University Graduate School, Tokushima, 770-8503, Japan, [2]Department of Digestive and Pediatric Surgery, Institute of Biomedical Sciences, Tokushima University Graduate School, Tokushima 770-8503, Japan, [3]R&D Group, Olympus Corporation, Hachioji, Tokyo 192-8512, Japan, [4]Graduate School of Pharmaceutical Sciences, The University of Tokyo, Tokyo 113-0033, Japan, [5]Laboratory of Veterinary Pathology, Tokyo University of Agriculture and Technology, Fuchu, Tokyo 183-8509, Japan.

***Correspondence**: Tetsuji Takayama

Department of Gastroenterology and Oncology, Institute of Biomedical Sciences,

Tokushima University Graduate School,

3-18-15, Kuramoto-cho, Tokushima, 770-8503, Japan.

Tel: +81-88-633-7124, Fax: +81-88-633-9235

E-mail: takayama@tokushima-u.ac.jp

Supplementary Results

Protein expression of candidate genes in human ACF tissues

Protein expression of the seven candidate genes (Glut-1, GSTP1-1, c-MET, β-catenin, Cadherin-1, SLC7A7, EGFR) in five human ACF tissue specimens were examined by immunohistochemistry. Representative results are shown in Supplementary Figure 2. Glut-1 stained strongly in the membrane of ACF cells. GSTP1-1 stained strongly, mainly in the cytosol of ACF cells. Moreover, Cadherin-1 and β-catenin were appreciably stained in ACF cells but were also stained in normal epithelia, indicating that protein expression by these genes in ACF was relatively weak. On the other hand, the staining signals for c-MET, SLC7A7 and EGFR were weak in ACF cells. Similar results were obtained in the remaining four ACF tissues.

Protein expression of GSTP1-1 and Glut-1 in rat ACF tissues

Expression of GSTP1-1 and Glut-1 proteins in five rat ACF tissues was examined by immunohistochemistry. Representative results are shown in Supplementary Figure 3. Glut-1 stained strongly in the membrane of ACF cells. GSTP1-1 also stained strongly, mainly in the cytosol of ACF cells. Similar results were obtained in the remaining four ACF specimens.

In vivo molecular detection of ACF in AOM-treated rats

We observed ACFs *in vivo* on the rectosigmoidal mucosa of five AOM-treated rats using a thin veterinary endoscope after enema administration of DNAT-Me. Representative images are shown in Supplementary Figure 4. ACF were clearly visualized at 10 min on the sigmoidal mucosa, then the signal became slightly attenuated at 20 min and almost disappeared at 30 min. Similar results were obtained in the remaining four rats. These results clearly suggest that the topical application of DNAT-Me is able to visualize rat ACF not only *in vitro* but also *in vivo*.

Ex vivo molecular detection of human adenoma and cancer

We performed *ex vivo* molecular imaging of each adenoma in five patients and each adenocarcinoma in seven patients by DNAT-Me. Representative lesions of adenoma and adenocarcinoma are shown in Supplementary Figure 5. A strong fluorescence signal was observed in the entire adenoma lesion although a partly dappled appearance was apparent. Likewise, a strong fluorescence signal was observed in the entire cancer lesion. Similar results were obtained in the remaining four adenomas and six cancers. Thus, human adenomas and cancers were clearly visualized *ex vivo* by DNAT-Me. These data support the theory of an ACF-Adenoma-Carcinoma sequence in colorectal carcinogenesis.

Supplementary Methods

Probe and primer sets for real-time PCR

The probe and primer sets for each human gene were obtained from Applied Biosystems (Foster City, CA). The following TaqMan gene expression assays were used: GSTP1 (Hs00943351_g1), Glut-1 (SLC2A1) (Hs00892681_m1), c-MET (Hs01565584_m1), β-catenin (ctnnb1) (Hs00355049_m1), SLC7A7 (Hs00909952_m1), Cadherin-1 (Hs01023894_m1), EGFR (Hs01076078_m1), Fzd1 (Hs00268943_s1), iNOS (NOS2) (Hs01075529_m1), CD44 (Hs01075861_m1), COX-2 (Hs00153133_m1), c-KIT (Hs00174029_m1), EPHB3 (Hs00177903_m1), CD24 (Hs00273561_s1), Glut-4 (SLC2A4) (Hs00168966_m1), ADAM17 (Hs01041915_m1), PTGER2 (Hs00168754_m1), CDK4 (Hs00175935_m1), CathepsinB1 (CTSB) (Hs00947433_m1), GPX-2 (Hs01591589_m1) and 18S (Eucaryotic 18S rRNA) (4333760T) as an internal control.

Human ACF tissues for immunohistochemistry

ACF tissues were obtained by biopsy under magnifying colonoscopy from five patients with colorectal adenoma or cancer, as described previously[13,14,39]. The specimens were fixed in 10% formalin, embedded in paraffin, and sliced into 5-μm sections for immunohistochemistry, as described previously[30].

Rat ACF tissues for immunohistochemistry

Azoxymethane (AOM) was administered to five male F344 rats subcutaneously at a dose of 15 mg/kg once a week for two weeks. The animals were sacrificed at eight weeks, and the colorectum was removed and fixed in 10% formalin. After ACF observation under a stereomicroscope, the colorectal mucosa including ACF was excised, embedded in paraffin, and sliced into 4-μm sections for immunohistochemistry as well as for haematoxylin and eosin stain, as described previously[41].

Immunohistochemistry

Immunohistochemical staining of human ACF tissues was performed using a streptavidin-biotin peroxidase method with labeled streptavidin-biotin (Dako, Tokyo, Japan), as described previously[43]. Rabbit anti-human glutathione S-trasnsferase P1-1 (GSTP) polyclonal antibody (MSA-102, Assay Designs Inc., Ann Arber, MI), rabbit anti-human Cadherin-1 polyclonal antibody (sc-7870, Santa Cruz Biotechnology Inc., Santa Cruz, CA), rabbit anti-human glut-1 polyclonal antibody (ab115730, Abcam, Cambridge Science Park, UK), rabbit anti-human c-MET polyclonal antibody (ab 51067, Abcam), rabbit anti-human β-catenin polyclonal antibody (sc-7199, Santa Cruz Biotechnology Inc.), rabbit anti-human SLC7A7 polyclonal antibody (HPA036227, Sigma-Aldrich Co., St Louis, MO), and rabbit anti-human epidermal growth factor receptor (EGFR) polyclonal antibody (sc-03. Santa Cruz Biotechnology, Inc.) were used as primary antibodies.

Immunohistochemistry for GSTP1-1 and Glut-1 in rat ACF tissue was also performed using the streptavidin-biotin peroxidase method with labeled streptavidin-biotin (Dako). Rabbit anti-human GSTP1-1 polyclonal antibody (MSA-102, Assay Designs Inc.) and rabbit anti-human glut-1 polyclonal antibody (Abcam, Cambridge Science Park), which crossreacts with rat counterparts, were used as primary antibodies.

In vivo molecular imaging of ACF in AOM-treated rats

AOM was administered subcutaneously to five male F344 rats, as described above. At eight weeks, the rats were anesthetized by inhalation of 3% isoflurane. The colorectum was cleaned by saline enema and infused with 3 mL DNAT-Me (200 μM). A rigid miniature probe (AE-R16150, AVS Co.,Ltd., Tokyo, Japan) was inserted into the colorectum, and the colorectal mucosa was carefully observed. The fluorescence image was monitored chronologically using a prototype fluorescence imaging system that was developed by modifying a veterinary endoscopic system (Olympus VR Type 7142A, AVS Co.,Ltd.) with a blue excitation filter (WRATTEN Gelatin Filter No.47, blue 410-500nm, Kodak, Rochester, NY) and a yellow emission filter (WRATTEN Gelatin Filter No.12, yellow 510+, Kodak).

Ex vivo molecular imaging of human adenoma and cancer

Five patients with adenomas (three patients with sigmoid colon adenomas and two patients with rectal adenomas) were enrolled. The average age and male-to-female ratio were 58.7 ± 14.1 years and 3:2, respectively. Colorectal adenomas were removed by endoscopic mucosal resection. The specimens were fixed on rubber, washed once with phosphate-buffered saline (PBS), and sprayed with 10 mL DNAT-Me (200 μM) directly onto the lesions. Similarly, seven patients with advanced CRC were enrolled. Patient characteristics are described in Supplementary Table. After surgical resection, colorectal tissues including cancer were washed

with PBS and sprayed with 20 mL of DNAT-Me (200 μM), as described in the text. They were observed using a fluorescent imaging system (Supplementary Fig. 7).

Ethics

This study was approved by the institutional review board of Tokushima University Hospital (Tokushima, Japan), as described in text. All human samples were obtained from patients who provided written informed consent. Animal experiments were performed according to the guidelines for animal experiments in Tokushima University Graduate School (Tokushima, Japan).

References

43. Sato, M, et al. High antitumor activity of pladienolide B and its derivative in gastric cancer. Cancer Sci 105, 110-6 (2014).

Supplementary Figure Legends

Supplementary Figure 1

Levels of mRNA expression for Fzd1, iNOS, CD44, COX-2, c-KIT, EPHB3, CD24, Glut-4, ADAM17, PTGER2, CDK4, GPX2, CD44, cathepsin B1 (CTSB), and GPX-2 in ACF and normal epithelia. Total RNA was extracted from ACF tissue and reverse transcribed, and TaqMan assay was performed to quantitate mRNA levels for each gene. The *18S* gene was used as an internal control.

Supplementary Figure 2

Immunohistochemical analysis for Glut-1, GSTP1-1, Cadherin-1, β-catenin, c-MET, SLC7A7 and

EGFR in human ACF. Immunohistochemical staining was performed on 5-μm sections of ACF tissue using a labeled streptavidin-biotin (LSAB) method. Serial sections are shown from a representative ACF specimen. Original magnification 100 x.

Supplementary Figure 3

Immunohistochemical analysis for Glut-1 and GSTP1-1 in rat ACF. Immunohistochemical staining was performed on 4-μm sections of ACF tissue using the LSAB method. Original magnification 100 x.

Supplementary Figure 4

In vivo molecular detection of ACF with DNAT-Me in the azoxymethane-treated rat carcinogenesis model. The colorectum in the rat was cleaned by saline enema and infused with 3mL DNAT-Me (200μM). Colorectal mucosa was chronologically observed using a rigid miniature probe. (A) White light, (B-D) fluorescence imaging at 10, 20, and 30 min respectively.

Supplementary Figure 5

ACF-Adenoma-carcinoma sequence. ACF (A), adenoma (B) and carcinoma (C) observed by conventional colonoscopy. ACF (D), adenoma (E) and carcinoma (F) detected by molecular imaging with DNAT-Me. (G) A scheme of the ACF-adenoma-carcinoma sequence.

Supplementary Figure 6

Structure of the GST-activated fluorogenic probe DNAT-Me. DNAT-Me has almost no fluorescence activity. When glutathionylated, it shows strong fluorescence, as described previously[32].

Supplementary Figure 7

A prototype fluorescent imaging system for the observation of colorectal lesions. A conventional light source CLV-180 (Olympus Corp., Tokyo, Japan) was used with an excitation filter. Fluorescence signals detected by the rigid scope were split and sent to both fluorescent and color cameras. The distance between the tip of the rigid scope and the subject was fixed at 50 mm.

Supplementary Table. Baseline Characteristics of the patients for molecular imaging of ACF

Case	Sex	Age	Location of cancer	Stage	Operative procedure
1	F	87	Sigmoid colon	II	Sigmoidectomy
2	F	70	Sigmoid colon	IIIa	Sigmoidectomy
3	M	74	Transverse colon	IIIa	Transverse colectomy
4	M	50	Rectum	II	Low anterior resection
5	F	73	Sigmoid colon	II	Sigmoidectomy
6	M	73	Rectum	IIIa	Low anterior resection
7	F	58	Sigmoid colon	II	Sigmoidectomy

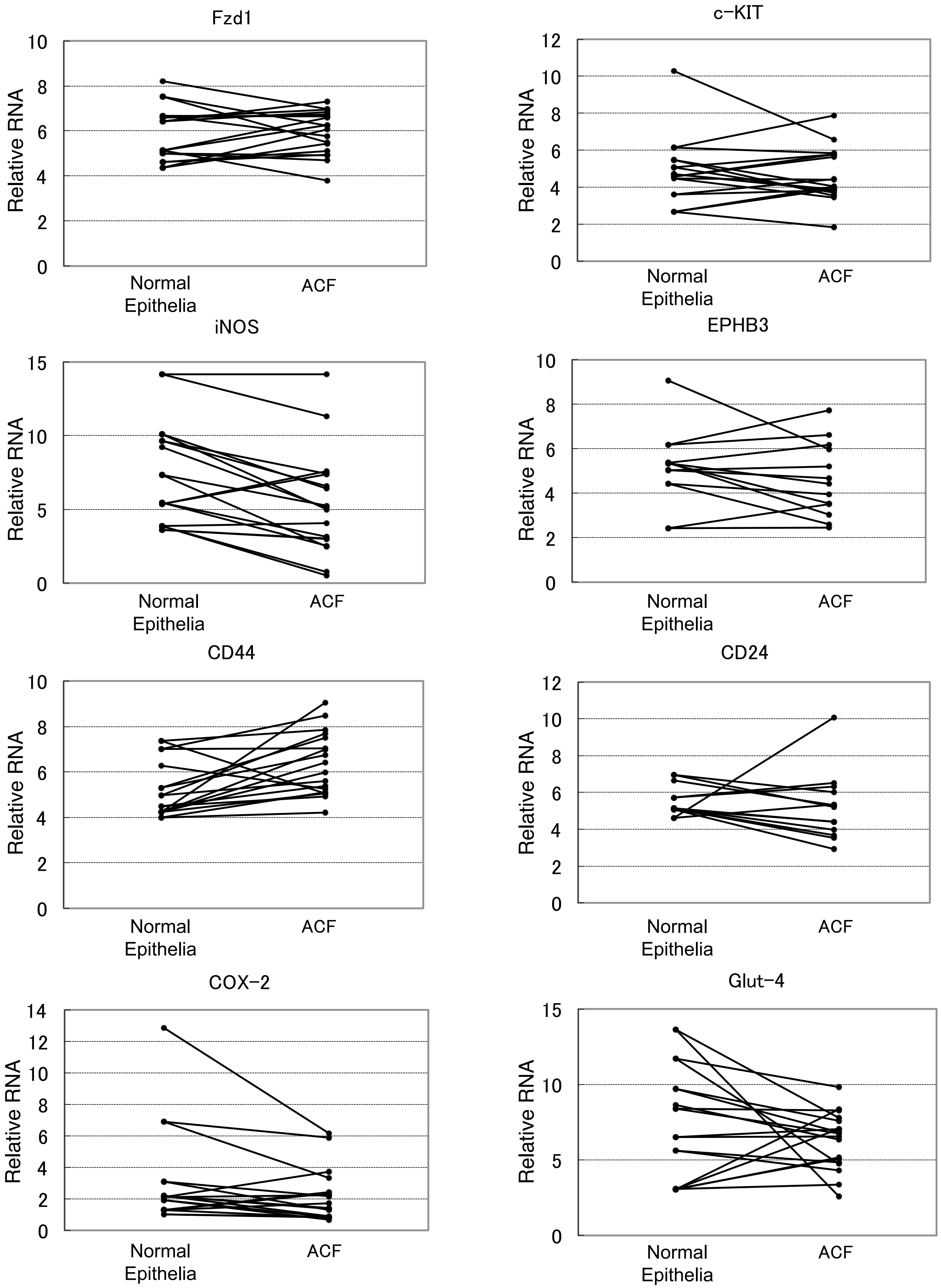

Supplementary Figure 1

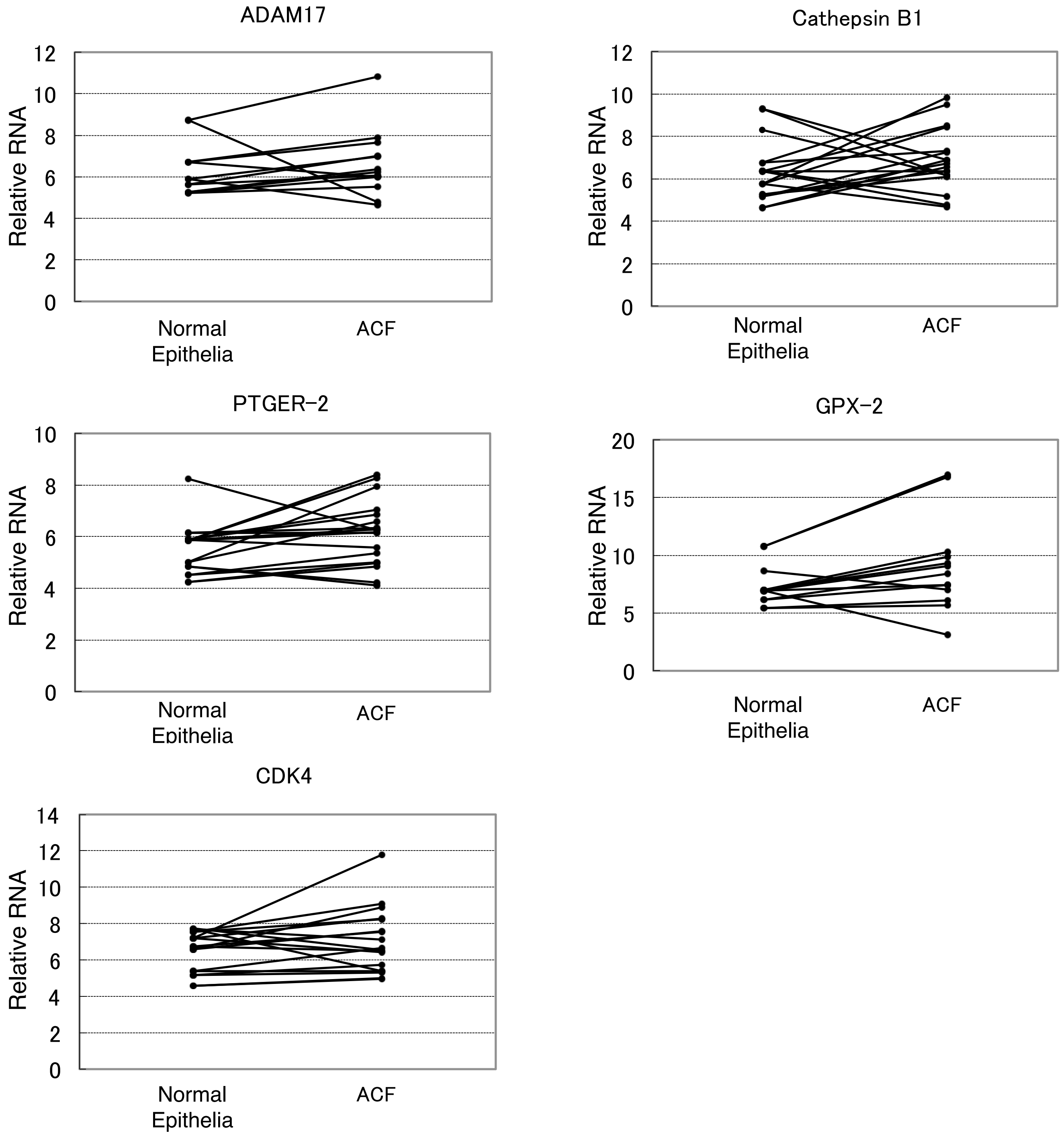

Supplementary Figure 1 continued

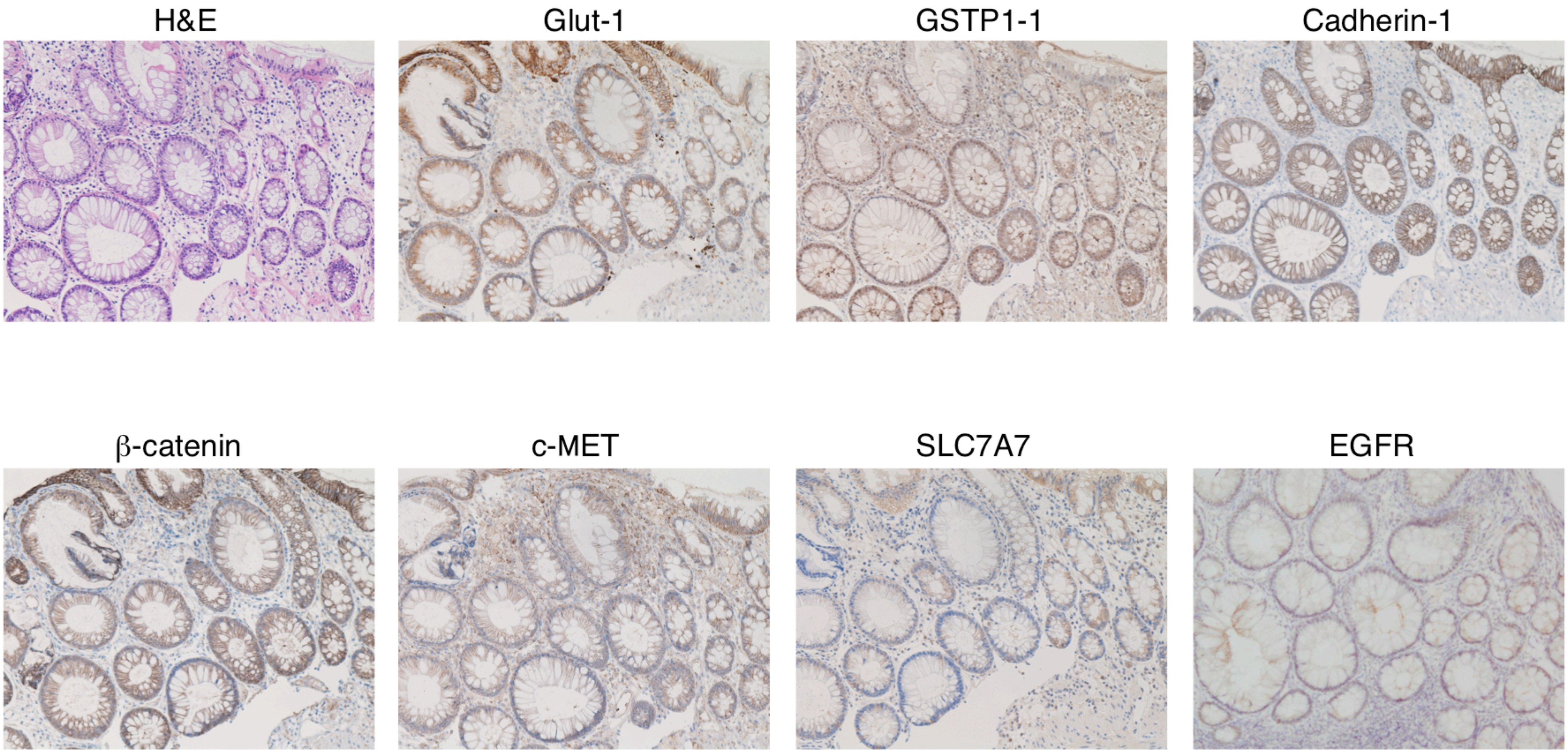

Supplementary Figure 2

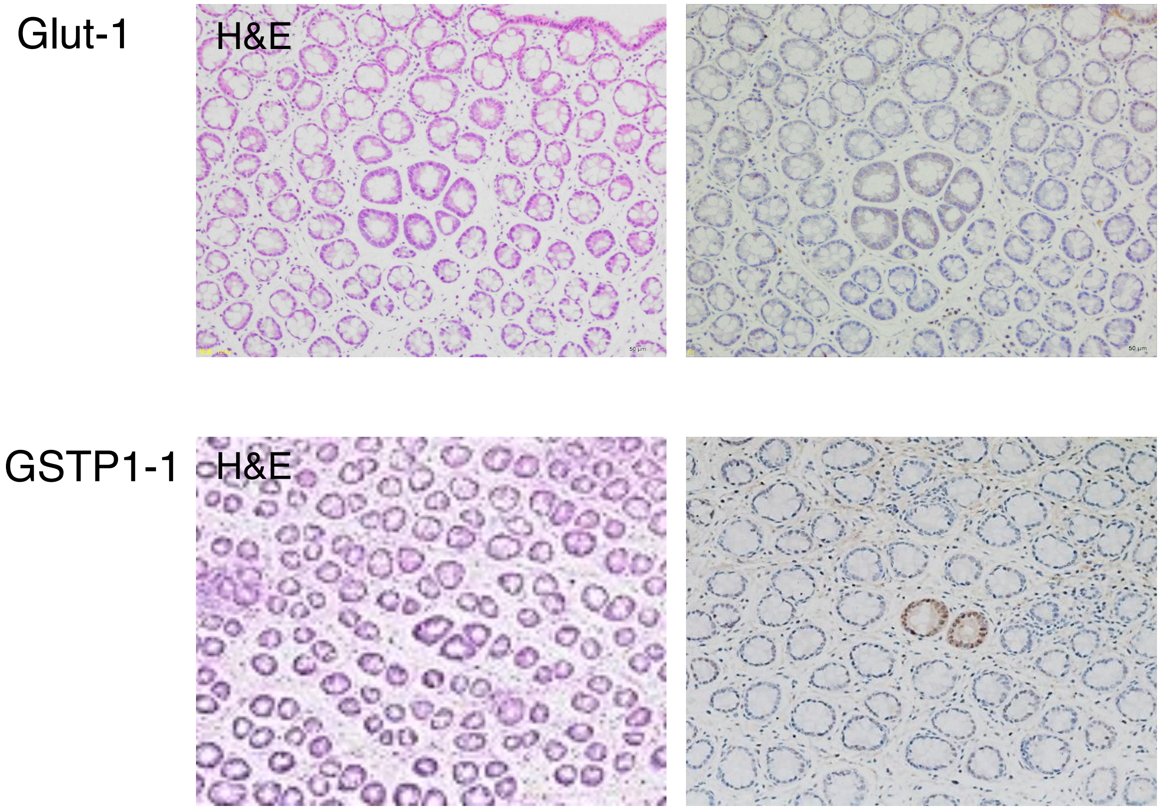

Supplementary Figure 3

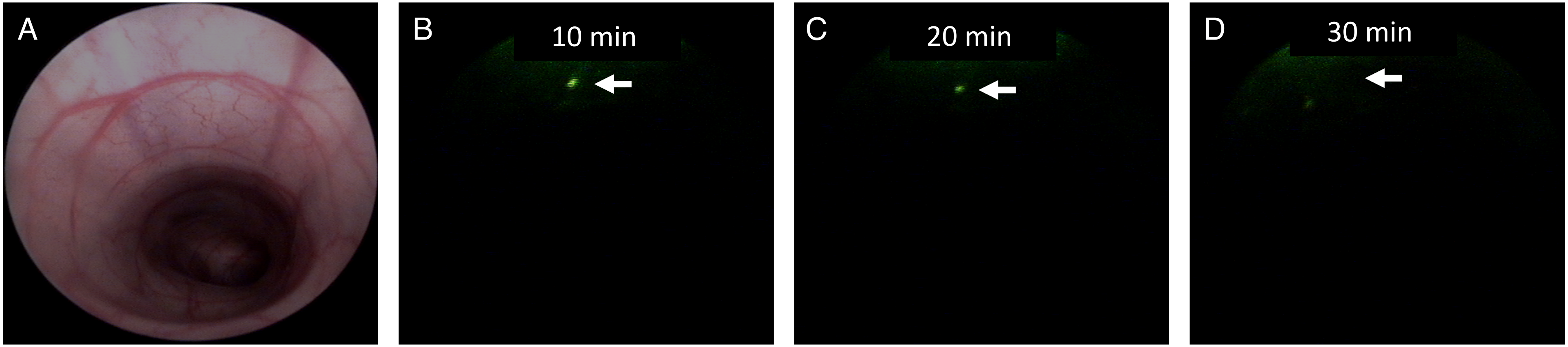

Supplementary Figure 4

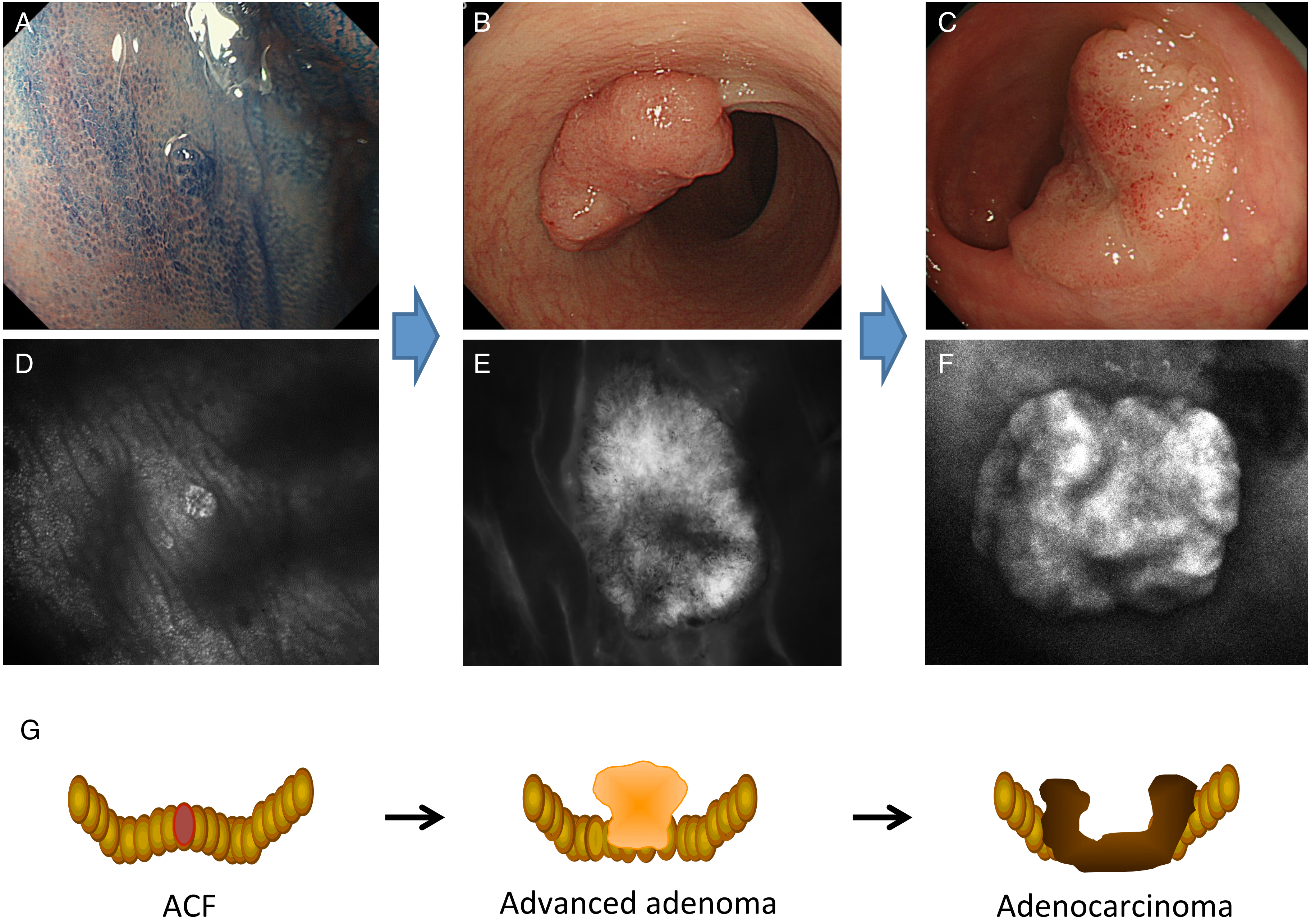

Supplementary Figure 5

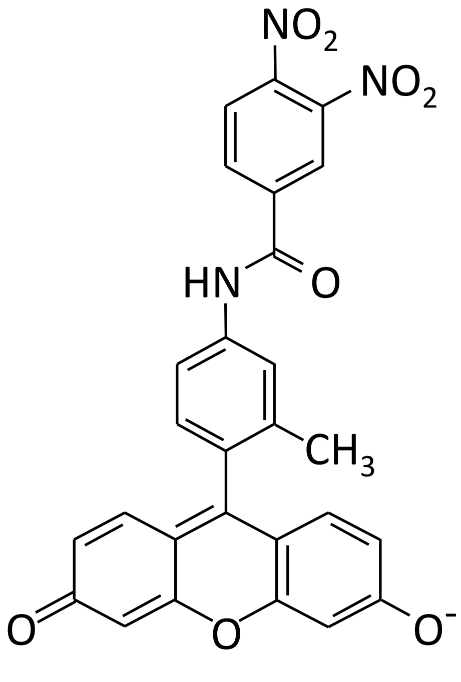

Supplementary Figure 6

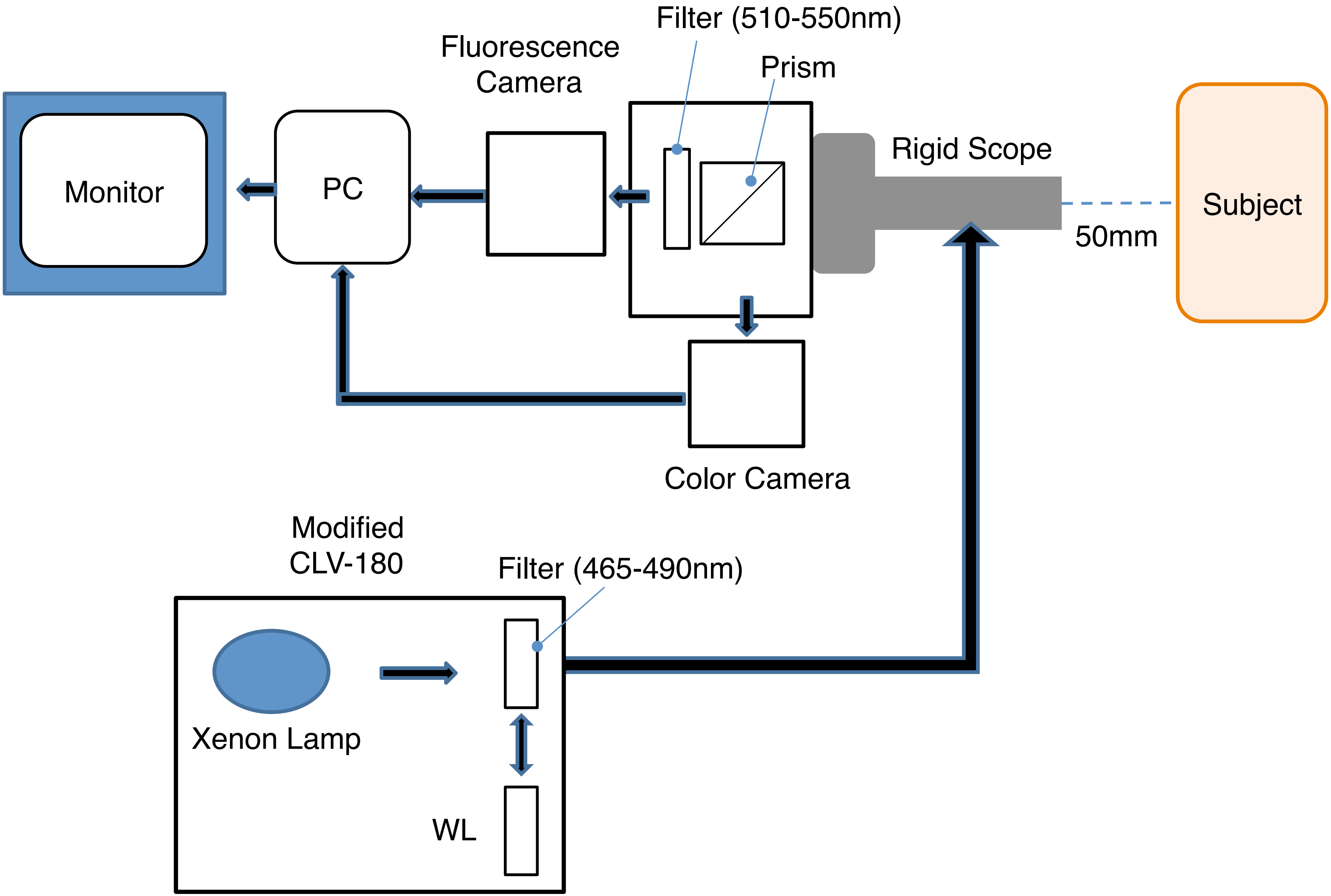

Supplementary Figure 7
